# Impact of antibacterial therapeutic agents on biofilm-tissue interactions in a 3D implant-tissue-oral-bacterial-biofilm model

**DOI:** 10.1038/s41598-025-03855-2

**Published:** 2025-05-30

**Authors:** Carina Mikolai, Kathrin Wöll, Muhammad Imran Rahim, Andreas Winkel, Christine S. Falk, Meike Stiesch

**Affiliations:** 1 Department of Prosthetic Dentistry and Biomedical Materials Science, Hannover Medical School, Carl-Neuberg-Str. 1, 30625 Hannover, Germany; 2 Lower Saxony Centre for Biomedical Engineering, Implant Research and Development (NIFE), Stadtfelddamm 34, 30625 Hannover, Germany; 3 Institute of Transplant Immunology, Hannover Medical School, Carl-Neuberg-Str. 1, 30625 Hannover, Germany

**Keywords:** 3D cell culture model, Multispecies biofilms, Antibiotics, Cytokines, Histology, Peri-implantitis, Biofilms, Cytokines, Target validation

## Abstract

Bacterial biofilms on dental implants can lead to peri-implant infections and demonstrate a remarkable ability to evade host immunity and resist antibiotics. Advanced in vitro models, such as the three-dimensional implant-tissue-oral-bacterial-biofilm model (INTER_b_ACT), are essential to evaluate antibiofilm efficacy. The INTER_b_ACT model, effectively reproduces the complex triangular interactions between an organotypic oral mucosa, an integrated implant and an oral multispecies biofilms, in the peri-implant situation. Here, we investigated the effect of antibacterial agents (chlorhexidine, amoxicillin, ciprofloxacin, doxycycline, and metronidazole) on biofilm-tissue interactions in the INTER_b_ACT model. While the antibacterial interventions had no effect on biofilm volume, all agents decreased the proportion of viable bacteria, underscoring their effect on bacterial viability despite biofilm resilience. Biofilm exposure to untreated tissues caused epithelial damage, whereas all antibacterial agents preserved epithelial integrity. However, the modulation of pro-inflammatory response differed between the various agents. All antibacterial treatments reduced hBD-2 and TIMP-1 levels. While doxycycline decreased IL-1β and CCL20, chlorhexidine lowered TNF-α level. In conclusion, the INTER_b_ACT model allowed the successful assessment of antibacterial efficacy, elucidation of biofilm resistance and characterization of inflammation during peri-implant tissue-biofilm interactions. This validation highlights the model’s potential as a platform for developing and evaluating new therapeutic strategies for peri-implant diseases.

## Introduction

Dental implants are used to restore oral functions and are among the most abundantly applied biomaterials, with approximately 1.3 million implants placed annually in Germany alone—a figure rising steadily due to demographic developments^1,2^. Despite their impressive success rate, implant failures can occur, largely due to peri-implant infections^3^. Implants are placed into a microbial-rich environment, and their success depends on proper integration with the surrounding soft and hard tissues^4^. A critical factor in this integration is the formation of a gingival soft-tissue seal, which serves as both a physical and immunological barrier to prevent bacteria from attaching to implant surfaces and forming multispecies biofilms^5^. Biofilm formation takes place in different stages. After initial pellicle formation on oral surfaces early colonizers such as Streptococcus spp., Actinomyces spp., and Veillonella spp. adhere. They establish multicellular bacterial communities and produce extracellular polymeric substances (EPS). Co-aggregation of additional bacterial species and growth lead to biofilm maturation. Late colonizers (mostly pathogenic species such as *Porphyromonas gingivalis*, *Treponema denticola* or *Tanerella forsythia)* can co-aggregate with early or intermediate colonizers (e.g., *Fusobacterium nucleatum*, Prevotella spp.), and drives the pathogenicity of the biofilm^6–10^. Under physiological conditions, the initial commensal biofilm is dominated by early colonizers, however, pathogenic bacteria, such as *P. gingivalis,* are also detectable at low level^11,12^. These biofilms maintain a symbiotic relationship with the host, promoting oral health through a balanced immune response^13,14^. However, various factors—including environmental stress, systemic disease, immune deficiency, and the presence of keystone pathogens (e.g., *P. gingivalis*)—can disrupt this equilibrium, leading to a shift in biofilm composition with larger amounts of pathogenic bacteria such as Porphyromonas spp., Prevotella spp., and Peptostreptococcaceae spp.^12,13,15^. As the prevalence of pathogenic bacteria increases, the host´s immune response intensifies, leading to inflammation of the peri-implant tissue, known as peri-implant mucositis or peri-implantitis^5^.

Peri-implant mucositis is characterized by reversible inflammation of peri-implant soft tissue and affects approximately 43% of the population^16^. If untreated, it can progress to peri-implantitis, a condition involving progressive bone loss around the implant^5^ and affecting 25% of implants five years after implant placement^17^. Therefore, professional management of peri-implant mucositis aims to effectively disrupt and remove bacterial biofilms from the implant to prevent the development of peri-implantitis^18,19^. The new S3 level clinical practice guideline (CPG) recommends the mechanical debridement as a primary treatment for peri-implant mucositis, whereby a time limited self-administration of oral antiseptic mouthwash, such as chlorhexidine, can be additionally used^20^. Due to concerns about patient’s health and potential public health implications, the S3 level CPG does not recommend the systemic use of antibiotics in patients with peri-implant mucositis. However, the application of antibiotics (such as amoxicillin, metronidazole, ciprofloxacin, or doxycycline) can reduce statistically significant the bleeding on probing (BOP), plague index (PLI), and pocket depth (PD) and can enhance the success rate of treatment without adverse side effects^20–26^. Therefore, the S3 level CPG recommends that there are no specific concerns for antibiotics as adjunctive treatment of peri-implant mucositis^20^. Moreover, as far as peri-implant mucositis can be seen as a precursor for peri-implantitis it is important to understand the mechanisms of antibiotics on the mucosa as the first line of defense of the immune system^27^. Antibiotics exert their effects by inhibiting bacterial growth (bacteriostatic) or killing bacteria (bactericidal). For example, the broad-spectrum antibiotic Amoxicillin disrupts cell wall synthesis, while doxycycline inhibits the ribosomal protein synthesis. Ciprofloxacin interferes with DNA replication by inhibiting DNA gyrase. Metronidazole, a prodrug, is activated under anaerobic conditions through microbial reduction of its nitro group, leading to DNA strand breaks in anaerobic pathogens. Consequently, antibiotics use various mechanisms to attack bacteria^28^. However, controlling peri-implant infections with antibacterial therapy remains challenging, as the resilience of multispecies biofilms often impedes treatment success.

In order to assess the clinical potential of antibacterial agents, it is imperative to use clinically relevant in vitro models that integrate the clinical phenotype of bacteria in multispecies biofilms and the critical cells of the host tissue in the same cultural environment. A model of the host–pathogen interaction that closely resembles that observed in clinical settings will likely result in more robust laboratory results that can be readily translated into clinical practice^29^. Traditionally, planktonic bacteria or two-dimensional (2D) monolayer cell cultures are used in in vitro evaluations of antibacterial agents. These studies fail to replicate the complex biofilm environment observed in vivo, where bacterial resistance is significantly higher^30–33^. In addition, antibacterial activity and cytocompatibility have primarily been investigated in separate experiments using either bacteria or human cells alone^34,35^. Furthermore, conventional 2D cell culture models lack the complexity needed to predict in vivo outcomes, as they do not replicate the three-dimensional (3D) structure and intercellular communication essential for simulating the peri-implant soft-tissue seal^36–39^.

In order to address these limitations, 3D peri-implant tissue models have been developed, which integrate various cell types, including epithelial cells and fibroblasts, and multispecies biofilms to simulate in vivo conditions more accurately^39^. The 3D-structure as well as the combination of different cell types influence various biological processes such as cell–cell communication, signaling, morphology, and cytokine secretion^40^. Therefore, these models overcome the limitations of 2D models and bridge the gap between laboratory research and clinical applications. 3D models closely replicate the host microenvironment and display a more accurate representation of the complex phenotype of native tissues^39,41^. Thus, 3D models can reliably be used to assess the biofilm-tissue interactions and the efficacy of antibacterial agents^29,39,42^. In addition to human tissue and bacterial biofilms, the implant also plays a crucial role in peri-implant diseases^43^. In order to reflect the complex triangular interactions between host cells, implants and biofilms, we have developed a unique 3D-implant-tissue-oral-bacterial-biofilm model (INTER_b_ACT)^44,45^. It consists of an organotypic oral mucosa, composed of stratified epithelium and fibroblasts combined with an integrated titanium implant, which is co-cultivated with an early commensal multispecies biofilm composed of *Streptococcus oralis*, *Actinomyces naeslundii*, *Veillonella dispar* and *Porphyromonas gingivalis*^45^. In this previous study, we have shown that the early peri-implant host-microbe interaction with a homeostatic situation and controlled immune response has been successfully reproduced in this model. After 48 h of co-cultivation, an enhanced immune response corresponding to the clinical situation has been observed, with a disruption of homeostasis and subsequent tissue damage^45^.

In the present study, we used the INTER_b_ACT model to evaluate antibacterial activity in a clinically relevant representation of the oral microenvironment. Therefore, the aims of this study were (i) to apply antibacterial agents (chlorhexidine, amoxicillin, ciprofloxacin, doxycycline, or metronidazole) in the INTER_b_ACT model for the first time and (ii) to investigate the effect of these antibacterial agents on multispecies biofilms, composed of *S. oralis*, *A. naeslundii*, *V. dispar*, *P. gingivalis*, on the organotypic peri-implant mucosa as well as on biofilm-tissue interactions.

## Results

### Increased dead proportion of biofilm after application of antibacterial agents in the INTER_b_ACT model

**Fig. 1:**
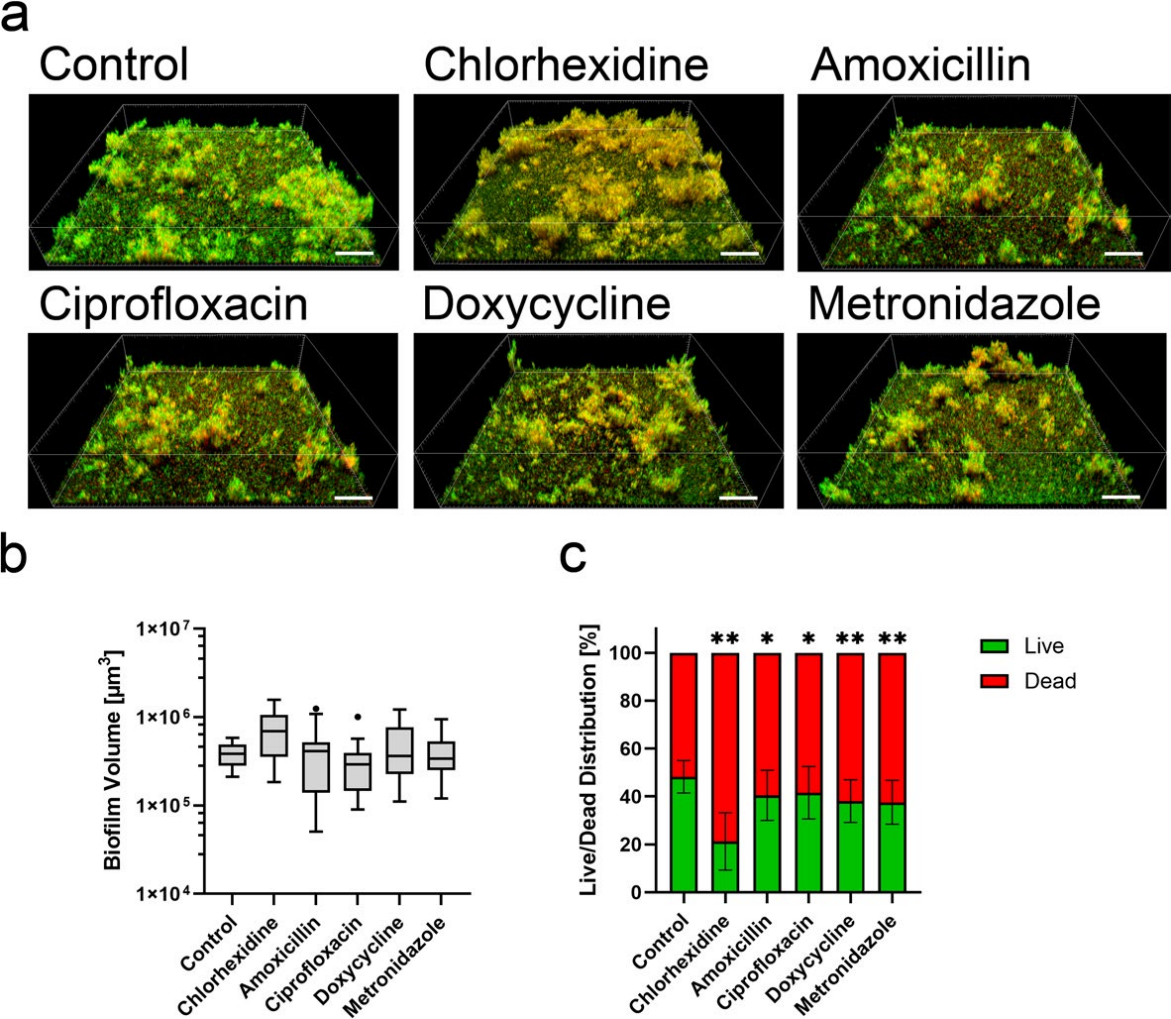
Increased dead proportion of biofilm after application of antibacterial agents. Multispecies biofilms were stained by live/dead following co-cultivation in the INTER_b_ACT model and application of antibacterial agents or water (control) as indicated. (**a**) Representative 3D images of live/dead-stained multispecies biofilms. Viable bacteria (intact membrane) are displayed in green, dead bacteria (damaged membrane) are displayed in red or orange. Scale bars: 50 µm. (**b**) The Turkey box plot shows the total biofilm volume, and (**c**) the bar graph the live/dead distribution (mean ± SD) of the biofilm, based on membrane integrity. Data represent six biofilms for each condition, with five measurements per sample. Statistical significance for biofilm volume was determined using the Kruskal–Wallis method, while two-way ANOVA was employed for live/dead distribution. *p < 0.05, **p < 0.01.

The effects of antibacterial treatment on host-biofilm interaction were evaluated by means of biofilm viability and composition. For this, multispecies biofilms were co-cultivated with an organotypic peri-implant mucosa and treated with either antibacterial agents or water as control. The biofilm biomass in the control group remained largely intact (Fig. 1a). Despite antibacterial treatment, biofilm biomass and structure within the multispecies biofilms closely resembled those of the control group (Fig. 1a). The biofilm volume also remained stable in the presence of chlorhexidine, doxycycline, and metronidazole, whereas amoxicillin and ciprofloxacin showed slight reduction of biofilm volume compared to the control (Fig. 1b). However, all antibacterial agents significantly increased the proportion of dead bacteria (damaged membrane) within the biofilms, analyzed by fluorescence-based membrane integrity staining (Fig. 1c).

### No substantial changes in biofilm composition after application of antibacterial agents

**Fig. 2:**
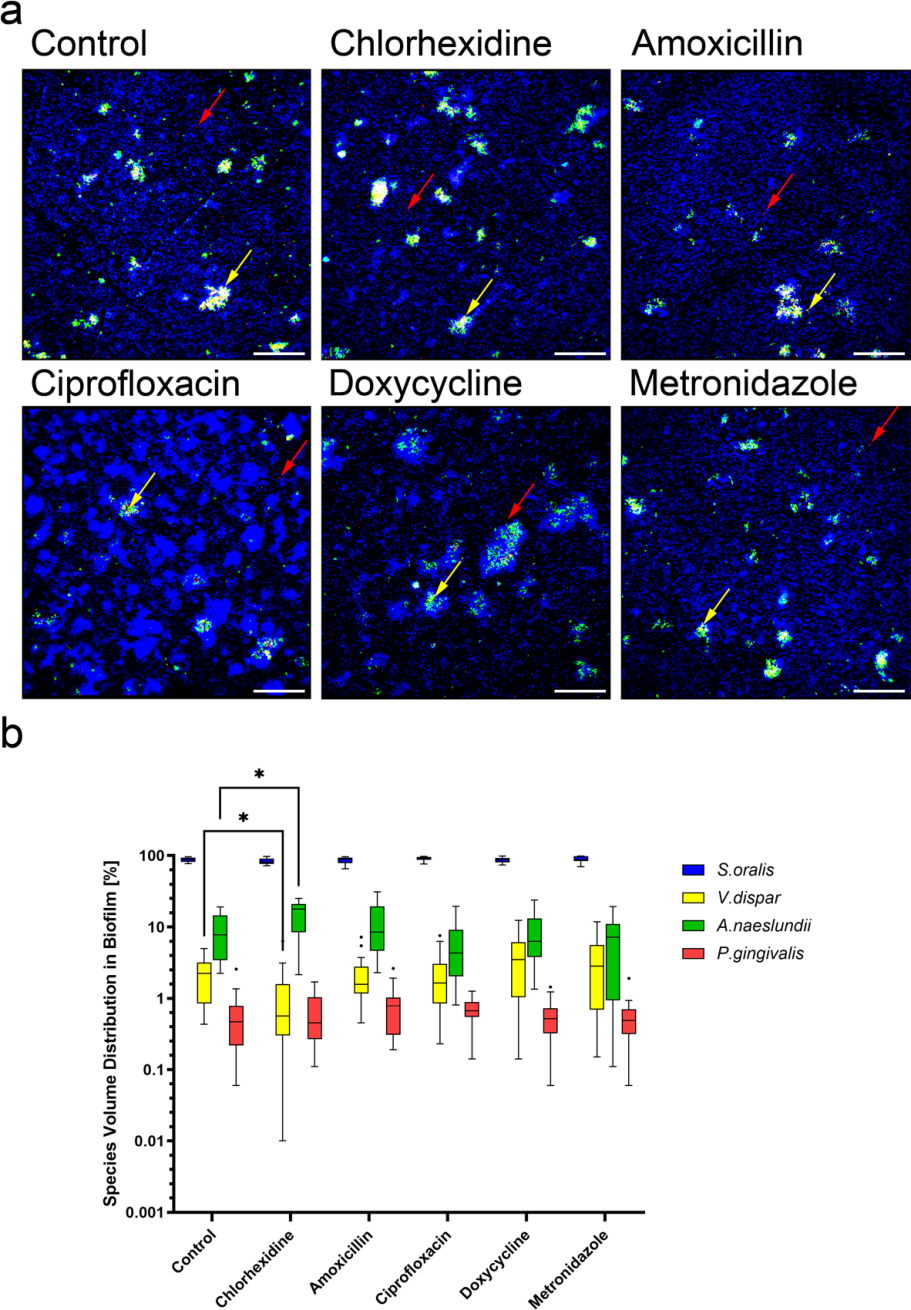
No substantial alteration in biofilm composition after application of antibacterial agents. Fluorescence in situ hybridization (FISH) staining of multispecies biofilms, which were co-cultivated with 3D tissues and treated with different antibacterial agents or water as a control. (**a**) Representative overlay images of FISH-stained multispecies biofilm. The individual bacteria were stained with 16S rRNA FISH probes: *S. oralis*—blue; *A. naeslundii*—green; *V. dispar*—yellow; *P. gingivalis*—red. Arrows indicate the respective species. Scale bars: 50 µm. (**b**) Tukey box plot shows the percentage volume proportion of each bacterial species in the multispecies biofilm. Data represent six biofilms for each condition, with four measurements per sample. Statistical significance was determined using two-way ANOVA. *p < 0.05, **p < 0.01.

We used fluorescence in situ hybridization (FISH) analysis to characterize the spatial organization and distribution of the four bacterial species. After application of the different antibacterial agents on multispecies biofilms cultivated in the INTER_b_ACT model, *S. oralis* and *A. naeslundii* were widely dispersed throughout the multispecies biofilm, while *V. dispar* and *P. gingivalis* were confined to microcolonies (Fig. 2a). The calculation of the relative volume proportions of each bacterial species showed that the most abundant species was *S. oralis*, followed by *A. naeslundii*, *V. dispar* and *P. gingivalis* in the control biofilms (Fig. 2b). While chlorhexidine disturbed the relation of the bacterial species and led to a significant reduction of *V. dispar* levels and increase of *A. naeslundii* proportion relative to controls, no statistically significant changes were observed for the other antibacterial agents compared to the control. Overall, the antibacterial treatments did not induce substantial changes in biofilm composition.

### Intact mucosal morphology after treatment with all antibacterial agents

**Fig. 3:**
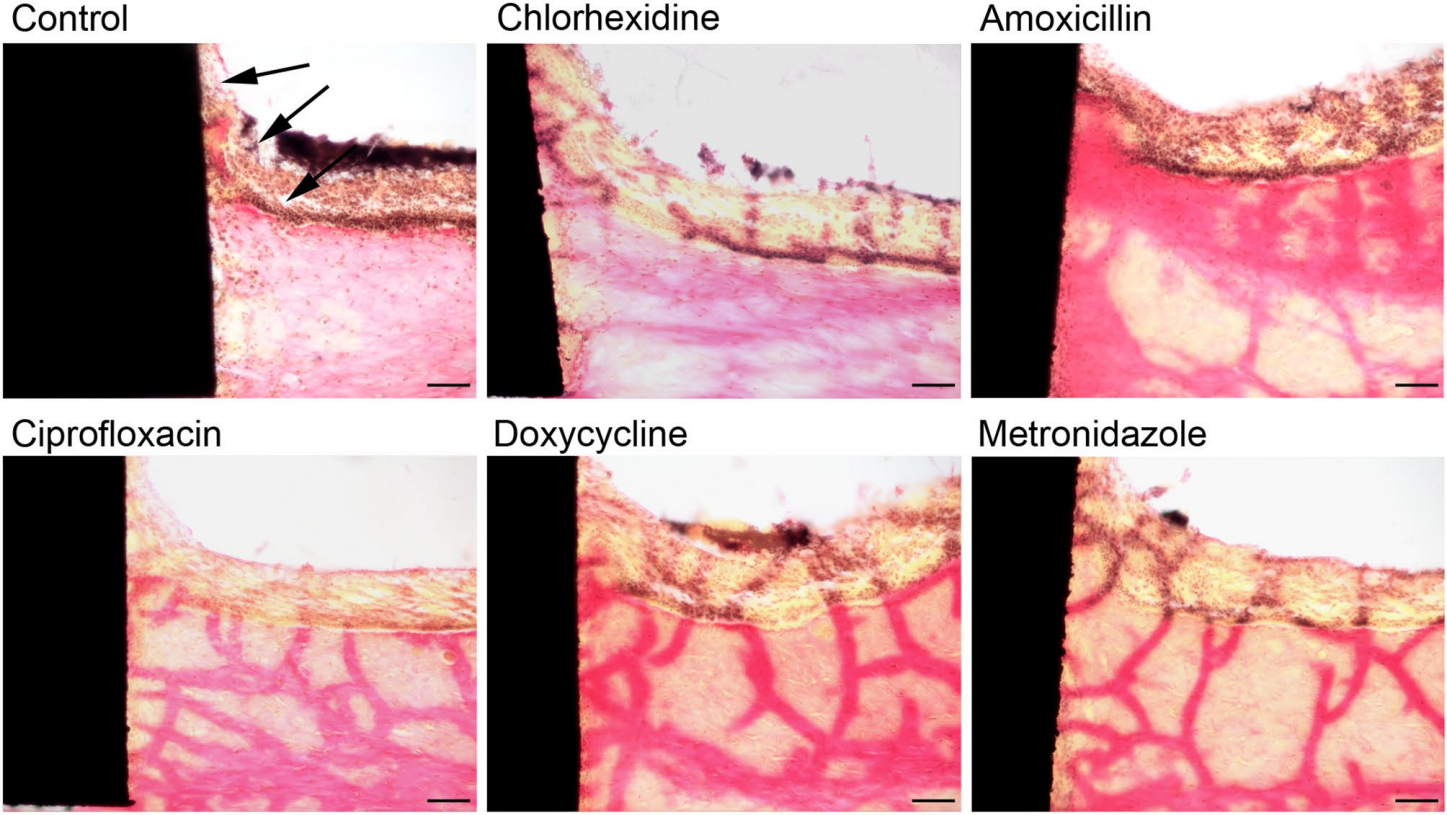
Application of antibacterial agents protects the mucosal morphology. Histological sections of the peri-implant mucosa after application of antibacterial agents or water as a control. The ground sections were stained according to van Gieson. The presented figures are representative of eight INTER_b_ACT models for each condition. Scale bars: 100 µm.

Histological analysis of the peri-implant mucosa following co-cultivation with multispecies biofilms was conducted to evaluate the role of antibacterial agents in preserving the integrity of peri-implant mucosa during tissue-biofilm interactions. The oral mucosa consisted of a multi-layered epithelium supported by connective tissue, composed of collagen-embedded human gingival fibroblasts. A titanium implant was integrated in this oral mucosa (Fig. 3). In the absence of antibacterial treatment, co-cultivation with multispecies biofilms caused damage to the epithelium with slight detachment from the implant (Fig. 3, black arrows), while the connective tissue containing fibroblasts remained intact and attached to the titanium. Treatment with chlorhexidine effectively prevented epithelial damage, leaving both the epithelial and connective tissues unaffected (Fig. 3, chlorhexidine). Likewise, the epithelial structure of the oral peri-implant mucosa remained well-preserved after treatments with amoxicillin, ciprofloxacin, doxycycline, and metronidazole (Fig. 3). Additionally, both treated and untreated tissues maintained a close and intact implant-mucosa interface, ensuring firm attachment to the implant surface (Fig. 3).

### Chlorhexidine and doxycycline reduced pro-inflammatory cytokine secretion

**Fig. 4:**
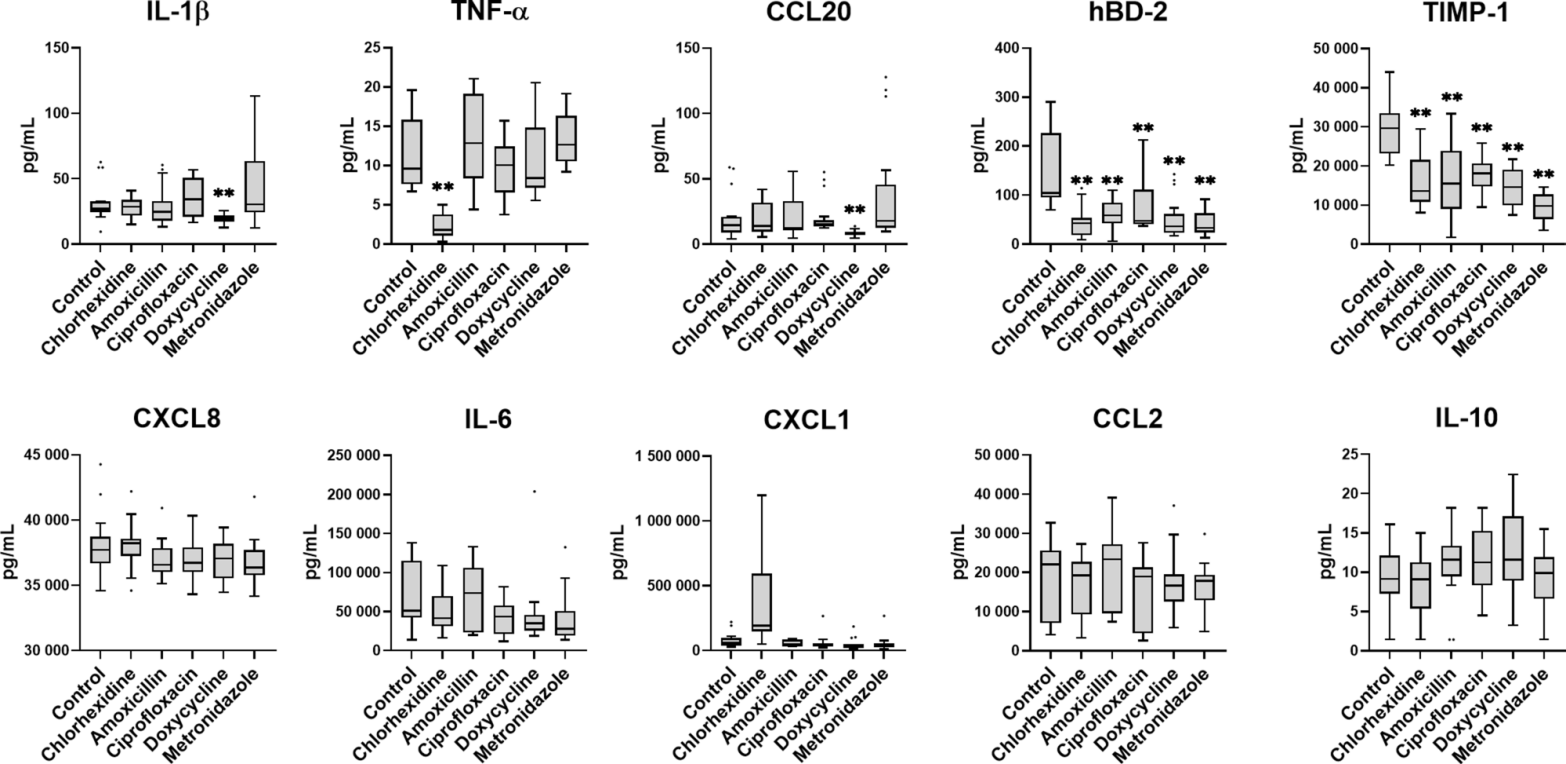
Reduced protein secretion after treatment with antibacterial agents. The concentrations of cytokines (IL-1β, TNF-α, IL-6, IL-10) and chemokines (CCL20, CXCL8, CXCL1, CCL2) were measured using Luminex-based multiplex technology, while hBD-2 and TIMP-1 levels were quantified by ELISA. Tukey box plots illustrate the measured data points (18 to 24) collected from six to eight individual INTER_b_ACT models for each experimental condition. Statistical significance was assessed using the Kruskal–Wallis method, *p < 0.05 and **p < 0.01. Abbreviations: IL-1β, interleukin-1β; TNF-α, tumor necrosis factor-α; CCL20, chemokine (C–C motif) ligand 20; hBD-2, human beta-defensin-2; TIMP-1, tissue inhibitor of metalloproteinase-1; CXCL8, chemokine (C-X-C motif) ligand 8 (interleukin-8); IL-6, interleukin-6; CXCL1, chemokine (C-X-C motif) ligand 1 (Gro-α); CCL2, chemokine (C–C motif) ligand 2 (MCP-1); IL-10, interleukin-10.

Since biofilm exposure to tissues is characterized by inflammatory cytokine production, we assessed the levels of IL-1β, TNF-α, CCL20, hBD-2, TIMP-1, CXCL8, IL-6, CXCL1, CCL2, and IL-10 in supernatants collected from the INTER_b_ACT model after 48 h of biofilm co-cultivation, with or without antibacterial treatments. Co-cultivation of the 3D tissues with multispecies biofilms induced the expression of multiple inflammatory cytokines (Fig. 4). The application of antibacterial agents significantly altered the protein secretion. All antibacterial agents significantly reduced the concentration of hBD-2 and TIMP-1. Chlorhexidine application led to a significant reduction of TNF-α in comparison to the control on the one hand and to an increase of CXCL1 on the other hand. The treatment with doxycycline resulted in a significant reduction of IL-1β and CCL20. None of the antibacterial agents influenced the levels of CXCL8, IL-6, CXCL1, CCL2, or IL-10. MMP-8 secretion was below the detection threshold of ELISA (data not shown). Overall, the pro-inflammatory reaction was also specifically influenced by both chlorhexidine and doxycycline, with a significant reduction in TNF-α or IL-1β, respectively.

### No changes in the gene expression after application of antibacterial agents

**Fig. 5:**
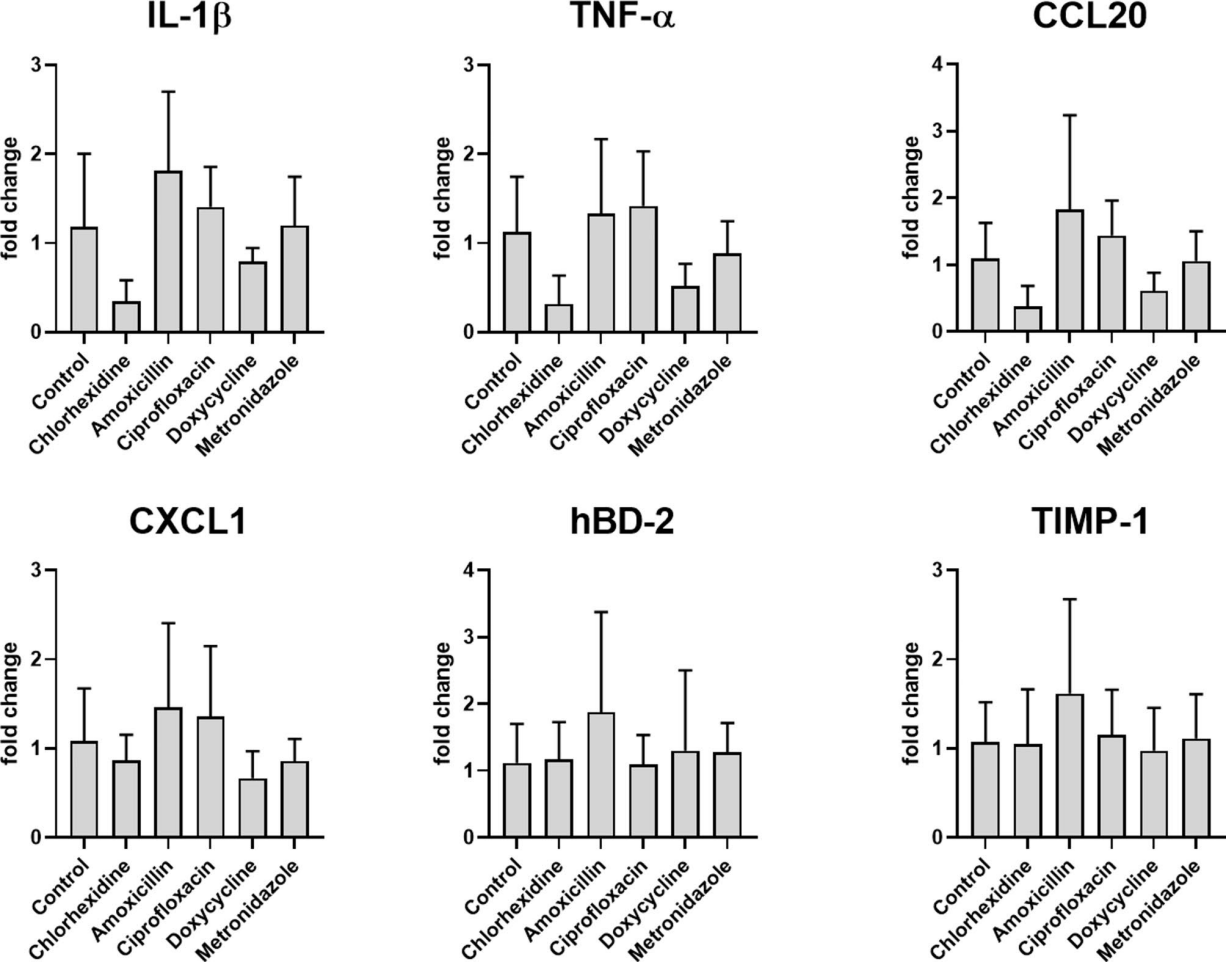
No significant alteration in pro-inflammatory gene expression after treatment with antibacterial agents. Gene expression of cytokines/chemokines (IL-1β, TNF-α, CCL20, CXCL1), hBD-2 and TIMP-1 after application of antibacterial agents. The gene expressions were analyzed using qRT-PCR. The bar graph illustrates the gene expression levels of cytokines/chemokines (IL-1β, TNF-α, CCL20, CXCL1), hBD-2, and TIMP-1 in comparison to untreated controls after administration of various antibacterial agents. Data points (n = 18) were collected from six INTER_b_ACT models under each experimental condition. The statistical significance was determined using the Kruskal–Wallis method, *p < 0.05 and **p < 0.01.

Since an altered protein secretion of IL-1β, TNF-α, CCL20, CXCL1, hBD-2, and TIMP-1 was observed after the application of antibacterial agents, the gene expression of these proteins was analyzed to determine whether regulation occurs at the genetic level. As a result, we observed that co-cultivation of the peri-implant mucosa with multispecies biofilms led to the expression of inflammatory genes, including IL-1β, TNF-α, CCL20, CXCL1, hBD-2, and TIMP-1 (Fig. 5). The inflammatory response in untreated control tissues was largely comparable to that observed in antibacterial-treated tissues. Chlorhexidine and doxycycline treatment resulted in a modest downregulation of IL-1β, TNF-α, and CCL20 compared to untreated control tissues. Overall, the administration of antibacterial agents did not induce statistically significant changes in the expression levels of IL-1β, TNF-α, CCL20, CXCL1, hBD-2, or TIMP-1.

## Discussion

Peri-implant mucositis and peri-implantitis are inflammatory conditions caused by multispecies biofilms, predominantly composed of anaerobic bacteria that progressively degrade the tissues supporting dental implants^19,46^. These biofilms are known to be resistant to antibiotic treatment due to their complex structure and bacterial diversity^47^. In order to evaluate the efficacy of antibacterial agents against these resilient biofilms and to analyze the underlying mechanisms, advanced 3D in vitro models that reproduce the complex triangular interactions between host tissue, implant, and biofilm are essential.

In this study, we successfully used the 3D implant-tissue-oral-bacterial-biofilm (INTER_b_ACT) model to analyze the effect of antibacterial agents on the tissue-biofilm interaction. The INTER_b_ACT model effectively simulates the dynamic interactions between the oral mucosa, dental implant materials, and oral multispecies biofilms and provides a clinically relevant platform for evaluating the bioactivity and biocompatibility of antibacterial agents^45^. In this model the antibacterial agents chlorhexidine, amoxicillin, ciprofloxacin, doxycycline, and metronidazole were tested, that are commonly used in the treatment of peri-implant diseases and exhibit various antibacterial mechanisms^48–51^. In this study, low concentrations similar to concentrations in the sulcus fluid were applied to reflect the clinical conditions^50,52–56^. This approach enabled the evaluation of not only the antimicrobial activity but also the impact on host-biofilm interactions.

Live proportions of multispecies biofilms were reduced by all antibacterial agents. They had, however, no effect on the biofilm volume and the biofilm architecture remained intact, highlighting the protective nature of the biofilm’s exopolysaccharide matrix and microbial diversity^57^. Similar resistance patterns have been reported in other studies involving subgingival biofilms containing *P. gingivalis* and *F. nucleatum* in organotypic models^58–60^. Moreover, this is in accordance with previous in vitro studies in which the antibacterial effect of the agents on multispecies biofilms have been investigated with similar concentrations^60,61^. In order to obtain a reduction of biofilm volume, higher concentrations would be necessary, far exceeding clinically applied antibiotic concentrations used in systemic or local therapies^62,63^. Despite the low concentrations in our study, all antibacterial agents were able to significantly increase the dead proportion of the multispecies biofilm. This could possibly indicate that the peri-implant mucosa in fact influences the biofilm and makes it more susceptible for antibacterial agents. A possible explanation for this are antimicrobial peptides, which are known to degrade the biofilm matrix that enhances the antibacterial agents’ efficacy^64^. The reduced viability of the biofilms following application of antibacterial agents observed here may also explain the antibacterial efficacy of the agents reported in clinical studies^25,48,51^.

Analysis of the bacterial composition in biofilms cocultured with peri-implant mucosa showed a reduced abundance of *V.* *dispar* and an increased abundance of *A.* *naeslundii* after application of chlorhexidine, while *S.* *oralis* and *P.* *gingivalis* remaining largely unaffected. *V.* *dispar*, a typical early colonizer in healthy oral microbiomes, has also been associated with disease^65,66^. Our previous findings have revealed that an increase in *V.* *dispar* proportions in the 3D model was associated with an increase in antimicrobial peptides, tissue breakdown products, and the presence of *P.* *gingivalis*, all of which influence microbial composition in clinical environments^45^. Therefore, treatment with chlorhexidine, which is commonly used against peri-implant biofilms, and the associated reduction of *V.* *dispar* may contribute to the management of peri-implantitis^67,68^. In contrast, *V.* *dispar* persisted after antibiotic treatments. This is due to its known resistance to metronidazole, penicillin, and piperacillin-tazobactam^69^. Notably, none of the antibacterial agents effectively targeted *P.* *gingivalis*, a keystone pathogen that drives dysbiosis through interactions with commensal bacteria^70^. This is in accordance with previous findings that the biofilm formation with early colonizers supports *P.* *gingivalis* survival, making its cells less susceptible to antibacterial agents^71,72^. Overall, while each antibacterial agent decreased the number of viable bacteria cells within the biofilm, the biofilm volume and the bacterial composition, particularly with regard to *P.* *gingivalis*, were not substantially altered, underscoring the challenges in combating these resilient biofilms.

In the present study, despite biofilm exposure, the organotypic peri-implant mucosa maintained its structural integrity, exhibiting a multi-layered epithelium supported by collagen-embedded fibroblasts, similar to our previous findings^44,45^. Untreated biofilms caused epithelial damage, while the underlying connective tissues remained intact. Although in case of a clinical diagnosed peri-implant mucositis the observed tissue damage is not restricted to the epithelium, the results are consistent with previous in vitro studies indicating that *P.* *gingivalis* and other biofilm components degrade structural proteins and cause downregulation of focal adhesion genes in oral mucosa, both accompanied with compromise of epithelial integrity^45,73–75^. Importantly, in the present study all antibacterial agents demonstrated good biocompatibility and were able to successfully preserve the epithelium. In contrast, in other full-thickness 3D models chlorhexidine mouthwash has demonstrated cytotoxicity, possibly due to the higher concentration of chlorhexidine (0.2%) used in those models^76^. Our results suggest that at the concentration employed here, all antibacterial agents were capable of maintaining the integrity of the peri-implant mucosa, even if the biofilm was not completely eradicated.

Inflammation plays a central role in the progression of peri-implant diseases^77^, and we have, therefore, measured inflammatory cytokine secretion and expression in the present study. The biofilm exposure led to the secretion of pro-inflammatory cytokines like IL-1β, TNF-α, and CCL20, along with other markers such as hBD-2, TIMP-1, IL-6, CXCL1, CXCL8, CCL2, and IL-10. These results with particularly increased IL-6, CXCL8, CXCL1, and CCL20 secretion correspond to the observations in reconstructed human gingiva models of other studies, which considered important intermediate colonizers, such as *F. nucleatum* or Prevotella ssp. with immune modulating capacity, in more complex saliva-derived commensal multispecies biofilms^12,78–80^. Following antibacterial treatment, hBD-2 and TIMP-1 levels were reduced in the present study. These markers are associated with inflammatory diseases and known to be elevated in peri-implantitis^81–84^. Doxycycline was particularly effective in significantly reducing CCL20 and IL-1β levels, while chlorhexidine significantly reduced TNF-α secretion suggesting potential roles in modulating the inflammatory response. This is supported by clinical studies, which have shown that both doxycycline and chlorhexidine are able to reduce IL-1β or TNF-α^85,86^. Thereby, the analysis of gene expression demonstrated that reduction of hBD-2, TIMP-1, CCL20, IL-1β and TNF-α by the antibacterial agents was only observed at the secretion level. Gene expression after antibacterial treatment remained unchanged in comparison to the control. Similar to this study, in a 3D oral mucosa model infected with oral bacteria pro-inflammatory cytokine/chemokine secretion (IL-1β, IL-6, and CXCL8/IL-8) has been significantly decreased upon chlorhexidine treatment compared to untreated controls^76^. Furthermore, an in vitro monoculture study has shown that tetracycline has significantly downregulated the mRNA expression level of TNF-α in *Aggregatibacter actinomycetemcomitans*-infected macrophages^87^. Although tetracycline was not examined in our study, these findings suggest that both chlorhexidine and certain antibiotics are effective in reducing TNF-α secretion, a key cytokine associated with peri-implant inflammation^88,89^. Consequently, all used antibacterial agents were able to protect the integrity of the peri-implant mucosa and reduced the secretion level of hBD-2 and TIMP-1. However, only chlorhexidine and doxycycline reduced the secretion of the key pro-inflammatory cytokines IL-1β or TNF-α, which both play important roles in the development of peri-implantitis^90,91^.

Although, the effects of antibacterial agents were successfully analyzed in the INTER_b_ACT model with indications for treatment success of peri-implant diseases, there remains further optimization potential with regard to improved transferability of the findings to the clinical situation. The applied multispecies biofilm lacks complexity with only four bacterial species and important bacterial species for inflammatory response missing (*F. nucleatum*) or underrepresented (*P. gingivalis*)^12,80^. Therefore, a further improved INTER_b_ACT model, with integration of complex patient-derived multispecies biofilm and immune cells in the organotypic mucosa, could enable investigations of antibacterial agents with even greater relevance for clinical applications. Nevertheless, a comparison of the effect of antibacterial agents in the INTER_b_ACT model reveals that all agents had a similar impact on multispecies biofilms (live/dead distribution), but a different effect on the inflammatory reaction of the tissue. Considering the biofilm alone would suggest that the choice of antibacterial agent used for the treatment of peri-implant infection does not influence the therapeutic outcome, since all antibacterial agents reduced the viable proportion of the biofilm. However, only chlorhexidine and doxycycline demonstrated a reduction of key cytokines associated with peri-implant infections, which may indicate a greater impact on treatment success^90,91^. This difference in effect on biofilms and on human tissues highlights the importance of interpreting new therapeutic strategies in a co-culture setting. This is in accordance with our previous study, showing that the antibacterial effect of silver-gold alloy nanoparticles has only been present in bacterial monocultures, but could not be detected in a co-culture with human epithelial cells^92^. These results suggest that the interaction between host and microbe can strongly influence the results of antibacterial testing. Furthermore, 3D co-culture models have a higher clinical relevance compared to 2D models^93^. Consequently, in vitro 3D co-culture models, such as our INTER_b_ACT model, are important tools for the investigation of new therapeutic strategies.

In conclusion, all antibacterial agents increased the dead proportion of bacteria, but failed to eradicate resilient biofilms containing pathogenic *P. gingivalis*. These findings highlight the need for more targeted and sophisticated therapies, potentially involving advanced drug delivery systems or sensor-actuator technologies, to fully address the challenges of biofilm-associated peri-implant diseases. The INTER_b_ACT model offers a powerful platform for testing such interventions, bridging the gap between in vitro research and clinical applications, and providing a more nuanced understanding of host–pathogen dynamics and therapeutic efficacy.

## Materials and methods

### Assembly of (3D) implant-tissue-oral-bacterial-biofilm model (INTER_b_ACT)

The three-dimensional model of per-implant mucosa was constructed in culture inserts (3414, Corning B.V. Life Sciences) in accordance with the methodology previously described^44,45^ (Figure S1). In brief, human gingival fibroblasts (HGFs, 4 × 10^5^ cells/model) (121 0412, Provitro GmbH) were embedded in a collagen type-I hydrogel mix (bovine type-I collagen (2 mg/mL, PureCol^®^, 5005-100ML, Advance Biomatrix) supplemented with FBS, L-glutamine (G7513, Sigma-Aldrich), 10 × DMEM (P03-01510, Pan-Biotech), and reconstitution buffer (2 mg/mL sodium bicarbonate, 2 mM HEPES, and 0.0062 N NaOH). Titanium disks (3 mm diameter, 2.3 mm height, grade 4, machined surface) were pre-colonized with HGF to prevent keratinocyte growth apically along the titanium disk. After 4 days, the pre-colonized titanium disks were integrated in HGF-loaded hydrogels. For this purpose, the hydrogels were punched with a 2.5 mm diameter biopsy punch and the titanium disks were placed in the resulting holes. Following a three-day incubation period, immortalized human oral keratinocytes (OKF6/TERT-2, 1 × 10^6^ cells/model) were seeded on top. After 2 days, models were raised to create an air–liquid interface and cultivated at 37 °C in a 5% CO_2_ humidified atmosphere for 15 days, with the objective of stimulating epithelial differentiation and stratification.

The reproducible, commensal multispecies biofilm consisting of *Streptococcus oralis* (ATCC^®^ 9811TM, American Type Culture Collection ATCC), *Actinomyces naeslundii* (DSM 43013, German Collection of Microorganisms and Cell Cultures), *Veillonella dispar* (DSM 20735), and *Porphyromonas gingivalis* (DSM 20709) was formed as previously described^45,61^. Briefly, the four bacterial species were pre-cultured at 37 °C under anaerobic conditions (80% N_2_; 10% H₂; 10% CO_2_) in brain heart infusion (BHI) medium (Oxoid), supplemented with 10 µg/mL vitamin K. The bacterial pre-cultures were mixed equally in BHI/vitamin K to achieve a final optical density (600 nm) of 0.01 for each species. The bacterial mixture was added on glass cover slips (18 mm in diameter, thickness 1, Thermo Scientific Menzel) in 6-well plates and cultivated for 48 h under static anaerobic conditions (less than 0.1% O_2_, 7–15% CO_2_) at 37 °C to form the multispecies biofilm.

As previously described, the peri-implant mucosa and the multispecies biofilm were combined to create the 3D-implant-tissue-oral-bacterial-biofilm model (INTER_b_ACT)^44,45^ (Figure S2). Briefly, the peri-implant mucosa models and the multispecies biofilms were washed separately with phosphate-buffered saline (PBS). The multispecies biofilms were then positioned on top of the titanium, which was integrated in the oral mucosa, and spacers placed adjacent to the tissue, with biofilm facing the oral mucosa. The INTER_b_ACT models were cultured after being submerged in co-culture medium (3:1 DMEM (P04-03591, Pan-Biotech) and Ham’s F-12 (P04-14559, Pan-Biotech), supplemented with 5 μg/mL insulin, 0.4 μg/mL hydrocortisone, and 2 × 10^−11^ M 5 triiodo-l-thyronine, 8 × 10^–5^ M adenine, 5 μg/mL transferrin, 10^–10^ M cholera toxin, 2 mM l-glutamine, 10% v/v FBS, 10% v/v BHI/vitamin K) at 37 °C in a humidified 5% CO_2_ atmosphere. After 24 h, the co-culture medium was replaced, the antibacterial agents were applied and the INTER_b_ACT models were further cultivated for 24 h.

### Application of antibacterial agents

The application of the antibacterial agents (0.01% chlorhexidine (4180, Caesar & Loretz GmbH), 15 µg/mL amoxicillin (A8523, Sigma-Aldrich), 2 µg/mL ciprofloxacin (29064, Santa Cruz), 2 µg/mL doxycycline (D9891, Sigma-Aldrich), or 15 µg/mL metronidazole (M1547, Sigma-Aldrich)) was conducted after a 24 h cultivation period of the INTER_b_ACT models (Figure S2). For this purpose, all antibacterial agents were prepared in water and sterile-filtered (0.22 µm pore size). The antibacterial agents were applied into co-culture medium between the mucosa and the biofilm, while water was added as a control. Subsequently, the INTER_b_ACT models were cultivated at 37 °C in a humidified 5% CO₂ atmosphere for further 24 h.

### Quantitative and qualitative biofilm analysis

After 48 h cultivation period in the INTER_b_ACT model, the multispecies biofilms were washed with PBS and stained with the LIVE/DEAD^®^ BacLight™ Bacterial Viability Kit (Life Technologies) as previously described^45^. The live/dead-stained biofilms were imaged using a confocal laser scanning microscope (CLSM, Leica TCS SP8, Leica Microsystems). Five z-stack images were obtained with a z-step size of 1 µm for each sample. Three-dimensional images were reconstructed, and the volume was calculated using the Imaris^®^ × 64 8.4 software package (Bitplane AG). The biofilm viability was analyzed by fluorescence-based membrane integrity staining. Viable (SYTO^®^9; green; intact membrane), dead (propidium iodide; red; damaged membrane), and colocalized (SYTO^®^9 + propidium iodide; green + red) parts of the biofilms were quantified. The colocalized part was defined as dead biofilm as well.

### Fluorescence in situ hybridization

After 48 h cultivation in the INTER_b_ACT model, the multispecies biofilms were stained using fluorescence in situ hybridization (FISH) as previously described^45,61^. Briefly, the fixed biofilms were permeabilized with lysozyme (1 µg/mL) for 10 min. Hybridization was performed with 4 µM of each 16S rRNA probe (Eurogentec) (Table S1). FISH-stained biofilms were imaged by CLSM. 3D-images were reconstructed and volumes of each bacterial species in the multispecies biofilm were calculated using Imaris^®^ × 64 8.4 software package.

### Histology of the peri-implant mucosa

The histological analysis of the per-implant mucosa was conducted following the cultivation in the INTER_b_ACT model, as previously described^44,45^. In brief, the peri-implant mucosa models were fixed and embedded in Technovit 9100. Tissue sections were sliced, ground down to a thickness of 22–36 µm and stained using the van Gieson method.

### Quantification of protein secretion

The supernatants of the INTER_b_ACT models were collected after 48 h of treatment. The concentrations of the following cytokines were quantified using a customized Human Chemokine Bio-Plex kit (Bio-Rad) with the Luminex-based multiplex technique, in accordance with the manufacturer’s instructions: CXCL1, IL-10, IL-1β, IL-6, CXCL8, CCL2, CCL20, and TNF-α. The concentrations were calculated using standard curves with five-parameter logistic (5-PL) regression curves (Bio-Plex Manager 6.0). Human β-Defensin-2 (hBD-2), tissue inhibitor of metalloproteinases-1 (TIMP-1), and matrix metalloproteinase-8 (MMP-8) were quantified using enzyme-linked immunosorbent assays (ELISAs). The ELISA kits were procured from PeproTech (hBD-2, TIMP-1) or R&D Systems^®^ (MMP-8) and used in accordance with the manufacturer’s instructions. The concentrations were calculated using a standard curve with a four-parameter logistic (4-PL) regression curve (Prism 8, GraphPad).

### RNA extraction and quantitative RT-PCR analysis

After the cultivation in the INTER_b_ACT model, tissues were stored in RNA later™ Solution (AM7020, Invitrogen) for gene expression analysis. The RNA was extracted using the RNeasy Mini Kit (74104, Qiagen) in accordance with the manufacturer’s instructions. In summary, the tissues were lysed with a microcentrifuge pestle in RLT buffer containing 1% β-mercaptoethanol, and the lysates were homogenized using a QIA shredder (79654, Qiagen). Subsequently, total cellular RNA was isolated using the RNeasy Mini Kit, without the on-column DNase digestion step. The quality and quantity of the total RNA were analyzed using the Bioanalyzer 2100 (RNA 6000 Nano Kit, 5067-1511, Agilent). A total of 500 ng of RNA was reverse transcribed with included DNase digestion using the QuantiTect^®^ Reverse Transcription Kit (205311, Qiagen) in accordance with the manufacturer’s protocol. Quantitative real-time polymerase chain reaction (qRT-PCR) was performed using TaqMan™ Fast Advanced Master Mix (4444557, Applied Biosystems, Thermo Fisher Scientific) and gene-specific TaqMan^®^ probes (Thermo Fisher Scientific) for IL-1β (Hs01555410_m1). TNF-α (Hs00174128_m1), CCL20 (Hs00355476_m1), CXCL1 (Hs00236937_m1), hBD-2 (Hs00823638_m1), or TIMP-1 (Hs99999139_m1) according to the manufacturer’s instructions. The level of the housekeeping gene glyceraldehyde-3-phosphate dehydrogenase (GAPDH, Hs99999905_m1) was used as an endogenous control. The relative fold change in gene expression was calculated using the comparative 2^−ΔΔCt^ method.

### Statistical analysis

All presented data were derived from three to four independent experiments, with technical duplicates for each condition. The statistical analysis was conducted using Prism 8 software (GraphPad, Software, San Diego, CA, USA). The Kruskal–Wallis test with Dunn’s correction was employed to ascertain the statistical significance of the differences between the control and the various antibacterial agent applications for the biofilm volume, protein secretion, and gene expression. The secretion of CXCL1 was excluded from the statistical analysis since 12 out of 18 measured values exceeded the detection level of the Luminex-based multiplex assay following chlorhexidine treatment. A two-way analysis of variance (ANOVA) with Bonferroni correction was employed to statistically analyze the comparison between the control and the antibacterial agents regarding the biofilm live/dead distribution and the species volume distribution. A p-value of less than 0.05 was considered statistically significant.

## Supplementary Information

Supplementary Information.

## Data Availability

The datasets generated and analyzed during the current study are available from the corresponding author on reasonable request.
