# Supplementary material for: Impact of antibacterial therapeutic agents on biofilm-tissue interactions in a 3D implant-tissue-oral-bacterial-biofilm model
Source: Sci Rep. 2025 May 30;15:18979. doi: 10.1038/s41598-025-03855-2 (PMC12125177; doi:10.1038/s41598-025-03855-2)
Supplement: Supplementary file 1 — Supplementary Information. [file 41598_2025_3855_MOESM1_ESM.pdf]

Supplementary Information

Impact of antibacterial therapeutic agents on biofilm-tissue interactions in a 3D implant-tissue-oral-bacterial-biofilm model

Carina Mikolai #, Kathrin Wöll #, Muhammad Imran Rahim #, Andreas Winkel, Christine S Falk, Meike Stiesch

#These authors contributed equally to this work

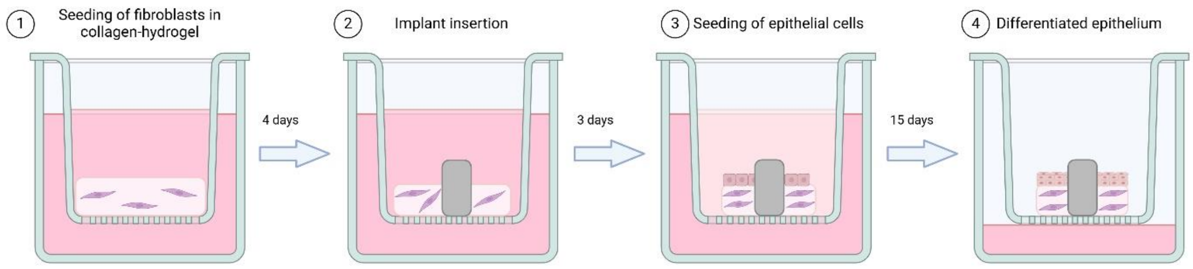

Figure S1: Schematic illustration of the assembly of 3D peri-implant mucosa model. 1. Human gingival fibroblasts were mixed with collagen type-I hydrogel mix and seeded in culture inserts. 2. After 4 days, the models were punched and the titanium implant were inserted in the hole. 3. After 3 days, human oral epithelial cells (OKF6/TERT2) were seeded on top of the models. 4. The models were raised to an air-liquid-interface and cultivated for 15 days to stimulate the epithelial differentiation and stratification. Created in BioRender. Winkel, A. (2025) https://BioRender.com/6hsfgt9

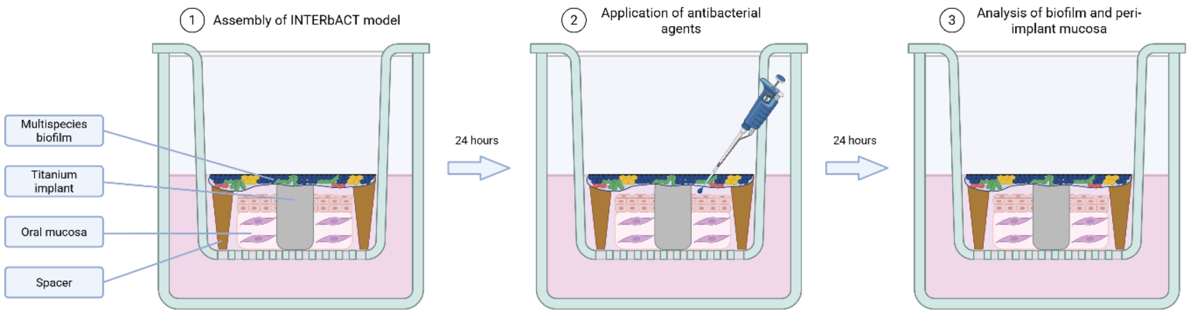

Figure S2: Schematic illustration of assembly of INTERbACT model and application of antibacterial agents. 1. After separately washing of multispecies biofilms and peri-implant mucosa models, biofilms were positioned on top of the titanium implant and on spacers placed adjacent to the tissue, with biofilm facing the oral mucosa. The INTERbACT models were cultivated after being submerged in co-

culture medium 2. After 24 hours, the antibacterial agents were applied into co-culture medium between the mucosa and the biofilm. 3. After 24 hours, both biofilms and peri-implant mucosae were analyzed by various methods. Created in BioRender. Winkel, A. (2025) https://BioRender.com/9xgjh1x

Table S1. Species-specific 16S rRNA probes for fluorescence *in situ* hybridization (FISH). The FISH stained biofilms were visualized using sequential imaging mode with two sequences. * first sequence; ** second sequence

**Probe**	**Species**	**Probe sequence [5'-3']**	**References**	**Label**	**Excitation [nm]**	**Emission [nm]**
MIT588	*S. oralis*	5' - ACA GCC TTT AAC TTC AGA CTT ATC TAA-3'	(Kommerein et al., 2017; Thurnheer, Gmur, & Guggenheim, 2004)	ALEXA Fluor®405	405*	413–477*
ANA103	*A. naeslundii*	5' - CGG TTA TCC AGA AGA AGG GG-3'	(Kommerein et al., 2017; Thurnheer et al., 2004)	ALEXA Fluor®488	488**	509–576**
VEI217	*V. dispar*	5' - AAT CCC CTC CTT CAG TGA- 3'	(Kommerein et al., 2017; Paster, Bartoszyk, & Dewhirst, 1998; Thurnheer et al., 2004)	ALEXA Fluor®568	552*	576–648*
POGI	*P. gingivalis*	5' - CAA TAC TCG TAT CGC CCG TTA TTC- 3'	(Kommerein et al., 2017; Sunde et al., 2003)	ALEXA Fluor®647	638**	648–777**

References

Kommerein, N., Stumpp, S. N., Müsken, M., Ehlert, N., Winkel, A., Häussler, S., . . . Stiesch, M. (2017). An oral multispecies biofilm model for high content screening applications. *PLoS ONE, 12*(3), e0173973. doi:10.1371/journal.pone.0173973

Paster, B. J., Bartoszyk, I. M., & Dewhirst, F. E. (1998). Identification of oral streptococci using PCR-based, reverse-capture, checkerboard hybridization. *Methods in Cell Science, 20*(1), 223-231. doi:10.1023/A:1009715710555

Sunde, P. T., Olsen, I., Gobel, U. B., Theegarten, D., Winter, S., Debelian, G. J., . . . Moter, A. (2003). Fluorescence in situ hybridization (FISH) for direct visualization of bacteria in periapical lesions of asymptomatic root-filled teeth. *Microbiology (Reading, England), 149*(Pt 5), 1095-1102. doi:10.1099/mic.0.26077-0 [doi]

Thurnheer, T., Gmur, R., & Guggenheim, B. (2004). Multiplex FISH analysis of a six-species bacterial biofilm. *Journal of Microbiological Methods, 56*(1), 37-47. doi:S016770120300246X [pii]
